# Analysis of Vehicle Collision on an Assembled Anti-Collision Guardrail

**DOI:** 10.3390/s21155152

**Published:** 2021-07-29

**Authors:** Juncheng Yao, Bo Wang, Yujie Hou, Liang Huang

**Affiliations:** 1 School of Water Conservancy Engineering, Zhengzhou University, Zhengzhou 450001, China; chengzi@gs.zzu.edu.cn (J.Y.); wangbo@zzu.edu.cn (B.W.); houyujie@zzu.edu.cn (Y.H.); 2 School of Civil Engineering, Zhengzhou University, Zhengzhou 450001, China

**Keywords:** car crash, guardrail, collision, bridge, traffic accident

## Abstract

Traffic accidents such as vehicle collisions with bridge guardrails occur frequently. These accidents cause damage to the driver and the vehicle as well as the bridge. A new type of assembled anti-collision guardrail is proposed in this study. LS-DYNA is a nonlinear display dynamic analysis software used to evaluate the safety of a new type of assembled anti-collision guardrail. A specific, numerically analyzed model of vehicle–guardrail collision is established using LS-DYNA. The energy distribution–time curve of the vehicle collision process is obtained. After comparison with measured data from the vehicle collision test, the model of vehicle–guardrail collision is verified as being correct. Based on this, we analyze the process of a vehicle collision on the assembled anti-collision guardrail. The result shows that the assembled anti-collision guardrail proposed in this paper can better change the trajectory of a moving vehicle and can prevent the vehicle from falling off the bridge. From the car body collision results, the assembled anti-collision guardrail for bridges proposed in this paper can reduce vehicle damage and can protect the driver effectively. From the analysis of the main girder stress on the bridge, an anti-collision guardrail installed on an existing bridge will not cause damage to the main girder during a collision. In order to study the influence of the four parameters on the anti-collision effect, we carried out a comparative calculation of multiple working conditions. The results show that the new type of assembled anti-collision guardrail has good protective performance under different working conditions.

## 1. Introduction

An effective guardrail system is the last defense that can protect the driver and passengers and that can return a vehicle to its correct driving direction when out of control to avoid traffic accidents caused by collisions between vehicles and guardrails. Guardrails can also guide traffic. Therefore, bridge guardrails are vital to safety and to transportation systems since bridges usually span rivers and valleys.

**Figure 1:**
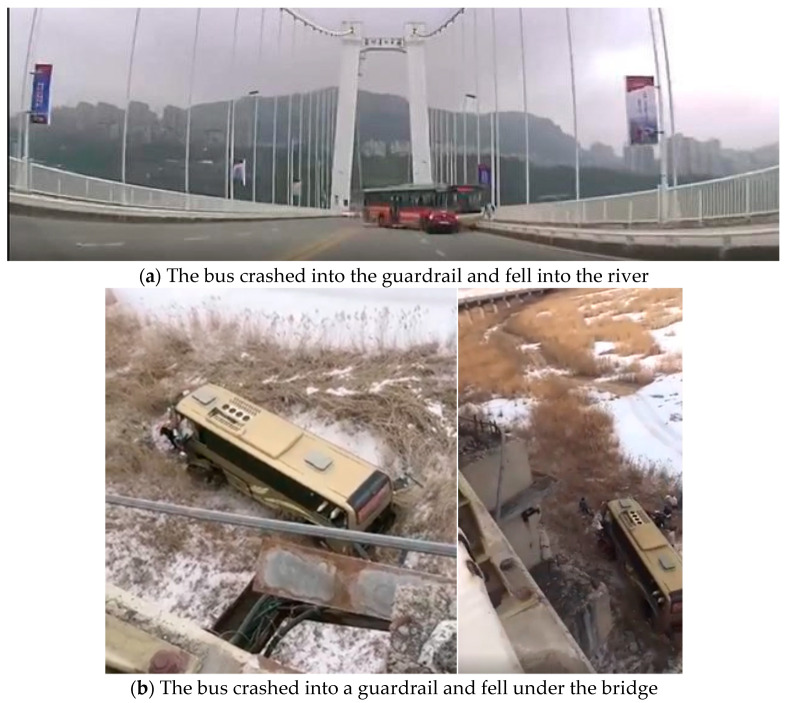
Vehicle collision with a guardrail of a bridge.

Investigations have shown that road traffic accidents cause 100 thousand deaths every year in China. One-third of fatal accidents are caused by vehicle collisions with guardrails [1]. On highways and bridges, serious traffic accidents are always caused by vehicle–guardrail collisions. In October 2018, a bus in Chongqing, China, fell into the river after crashing into the bridge guardrail, causing 13 deaths and 2 people to go missing, as shown in Figure 1a. In February 2021, a bus in Daqing City, China, crashed into a guardrail and fell under the bridge, causing two deaths and eight injuries, as shown in Figure 1b.

To protect the driver and passengers and to improve the guardrail collision performance, a series of studies using finite element simulation and on-site crash tests have been conducted. Ali O. Atahan performed a detailed finite element analysis on a 12 m long, downturned guardrail end treatment to fully evaluate its crashworthiness [2]. The response of a G4(1S) strong post steel w-beam guardrail system to pendulum impacts has been investigated [3]. Driemeier, L. studied the performance of a W-Beam guardrail system in accordance with Brazilian Standards with respect to the kinetic energy absorption and to the return angle of the vehicle to the road [4]. The crash performance of a strong post w-beam barrier that has sustained minor damage was introduced by Douglas J. Gabauer [5]. R.R. Neves studied the ability of various road barrier systems to reduce the overall damage in an accident [6]. The impact performance of a W-beam guardrail installed at various flare rates was studied by J.D. Reid [7]. An energy-absorbing end terminal was developed by N.M. Sheikh for use with the European box beam guardrail system [8]. An energy-absorbing end terminal was developed for use with the European box beam guardrail system. Some scholars studied the impact of guardrail construction tolerances and guardrail design parameters such as track installation height and wiring/non-wiring barriers on collision performance and guardrail safety [9,10]. A roadside guardrail system was anchored in the gravel or soil beside the road. Some scholars have studied the interaction between the guardrail column and the soil as well as the impact of the vehicle on the embankment slope when a vehicle crashes into the guardrail [11,12]. The United States has a design code for guardrails, such as the Roadside design guide [13] and the Manual for Assessing Safety Hardware [14]. After continuous study on guardrail collision performance, guardrail designs have been improved and enhanced. Since then, many improved guardrails have been developed to solve safety problems, especially on the roadside. A simple guardrail end treatment, called TWINY, designed particularly for use with a thrie-beam guardrail system, was developed [15]. A.O. Atahan developed a continuous motorcycle protection barrier system [16] and a new, high containment level (HCL) bridge rail to be used on bridges at high-risk locations [17]. The design optimization of a MASH TL-3 concrete barrier and the design optimization of a new W-beam guardrail for enhanced highway safety performance were introduced by H. Yin [18,19]. A.O. Atahan improved the crash test behavior of the New York Department of Transportation Portable Concrete Barrier (NYPCB) [20]. Additionally, other special guardrails have been studied, such as a portable water-filled road safety guardrail, which is used in temporary construction areas to improve road safety. An impact analysis of portable road safety barriers with external polymeric foam panels was introduced by R.B. Gover [21]. Thiyahuddin, M.I. introduced impact and energy absorption of a portable water-filled road safety barrier system fitted with foam [22] and the effect of a joint mechanism on the vehicle-redirecting capability of water-filled road safety barrier systems [23].

With the increase in motorization, more and more highways need be built. Therefore, establishing a new standard for guardrails to ensure the safety of the drivers and passengers has great significance.

At present, the bridge guardrail often used is embedded in the concrete of the bridge structure. We call this the traditional guardrail. When a vehicle collides with the guardrail, the main beam is easily destroyed. After the guardrail is damaged, it is difficult to replace it and the construction period is very long. To avoid the shortcomings of traditional guardrails, a new type of assembled guardrail is proposed to improve the collision performance of vehicles and to protect the driver and passengers effectively. Its goals are as follows:

(1) The guardrail can be assembled in construction, which can save construction time and can be replaced and maintained easily.
(2) When a vehicle crashes into the guardrail, the guardrail structure causes slight damage to the main body of the bridge.
(3) The guardrail meets the requirements of the specification.

Compared with the traditional concrete guardrail, the assembled guardrail can dissipate more energy, which is converted from the kinetic energy of the colliding vehicle. That means higher occupant safety and minimal damage to the vehicle. Therefore, the proposed assembled guardrail has better energy absorption performance and can reduce the damage to the bridge. The performance of the newly assembled guardrail under vehicle collision is described.

## 2. Design of a New Type of Assembled Guardrail

### 2.1. Design Conception

**Figure 2:**
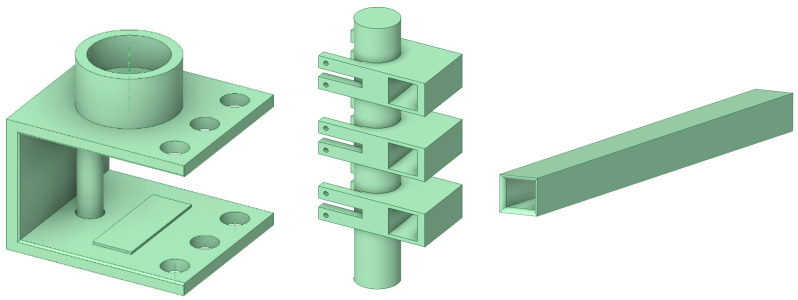
Schematic diagram of each component of the assembled bridge guardrail.

**Figure 3:**
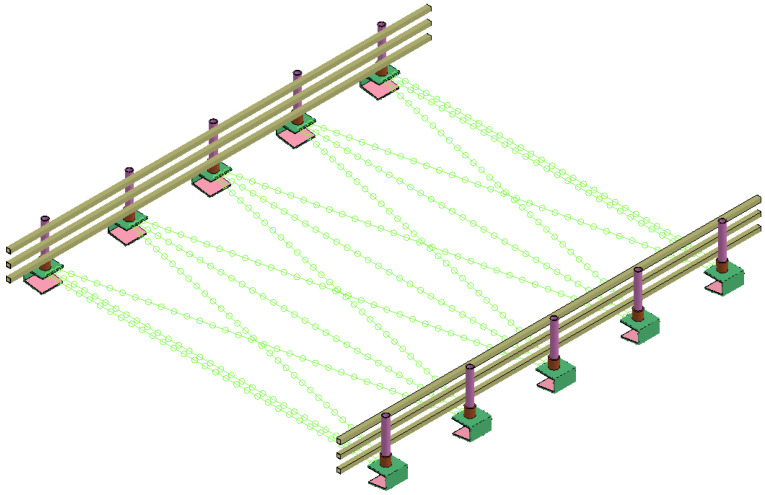
Three-dimensional drawing of the preassembled bridge guardrail.

The designed guardrail can be assembled in construction, which dramatically saves the time of guardrail replacement. Since the designed guardrail could reduce the impact of vehicle collisions, it can reduce the impact on the bridge’s main structure to a certain extent and can protect drivers and passengers. To improve the safety of the guardrail and to reduce the construction time, a new type of guardrail is proposed; it is composed of beams, a U-shaped base, columns, column sleeves, rubber pads, and steel strands, as shown in Figure 2 [24]. The crossbeam and the column are connected by M20 bolts, while the bottom of the column and the sleeve are connected by threads. The guardrail base is connected by a steel strand. The three-dimensional structure of the assembled bridge guardrail is shown in Figure 3, and the guardrail is composed of beams, a U-shaped base, columns, column sleeves, rubber pads, and steel strands. In a collision, the beams and columns collide with the vehicle, the collision force is transmitted to the U-shaped base through the column, and the deformation of the steel strand connected to the U-shaped base restricts the displacement of the U-shaped base. The rubber pad increases the friction between the U-shaped base and the bridge structure and reduces the damage to the U-shaped base to the bridge.

### 2.2. The Design Goal of the Assembled Guardrail

(1) The guardrail should be able to move laterally to increase the collision time, thereby reducing the instantaneous acceleration of the vehicle.
(2) The guardrail can be assembled in construction, which can save construction time and can be replaced and maintained easily.
(3) After a collision, the guardrail should control the vehicle’s direction effectively.

### 2.3. Working Mechanism of Assembled Guardrail

During the collision between a vehicle and the guardrail, the vehicle will suffer an impact force and friction force from the guardrail and the ground. Similarly, the decomposition force of the guardrail includes the impact load of the vehicle and the friction with the ground. When the lateral force is bigger than the friction with the ground, the collision guardrail segment and its adjacent segments move laterally, and the steel strand connecting the base of the guardrail column begins to deform. Due to deformation of the steel strands, and the friction between the guardrail base and the ground, certain parts of the vehicle collision energy can be dissipated, protecting drivers and passengers effectively.

LS-DYNA is used to simulate and analyze the collision process to verify the safety of the assembled guardrail system. In addition, a detailed finite element analysis is used to study the impact of the four main parameters on the collision performance of the guardrail: car mass, collision angle, car initial speed, and collision point.

## 3. Numerical Analysis Model

In this study, calculations are carried out based on a 2 × 15 m reinforced concrete box girder bridge with a span of 2 × 15 m in Xinzheng city of Henan Province in China. The original guardrail of the bridge is a steel pipe guardrail embedded in the base of the guardrail. The guardrail needs to be rebuilt due to accidents caused by vehicle collisions with the guardrail and subsequent accidents of falling off the bridge.

**Figure 4:**
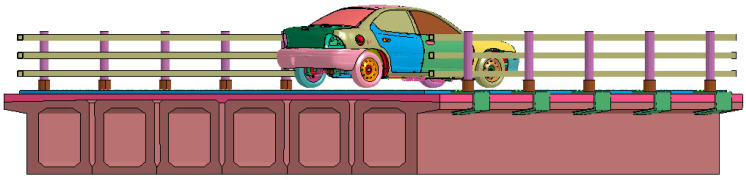
Car and bridge finite element 3D model.

**Figure 5:**
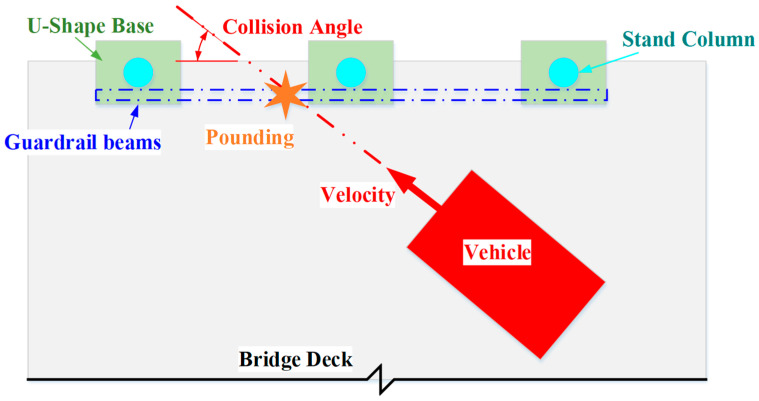
Collision diagram.

A middle span of 10 m is chosen for the calculations, and the box girder and the bridge deck are both C50 [25]. C50 is concrete with a compressive strength of 50 MPa. Figure 4 shows a 3D model of the collision between a car and the bridge guardrail. It can be seen from Figure 5 that the collision angle is the angle between the car velocity and the guardrail beam.

### 3.1. Bridge Model

**Figure 6:**
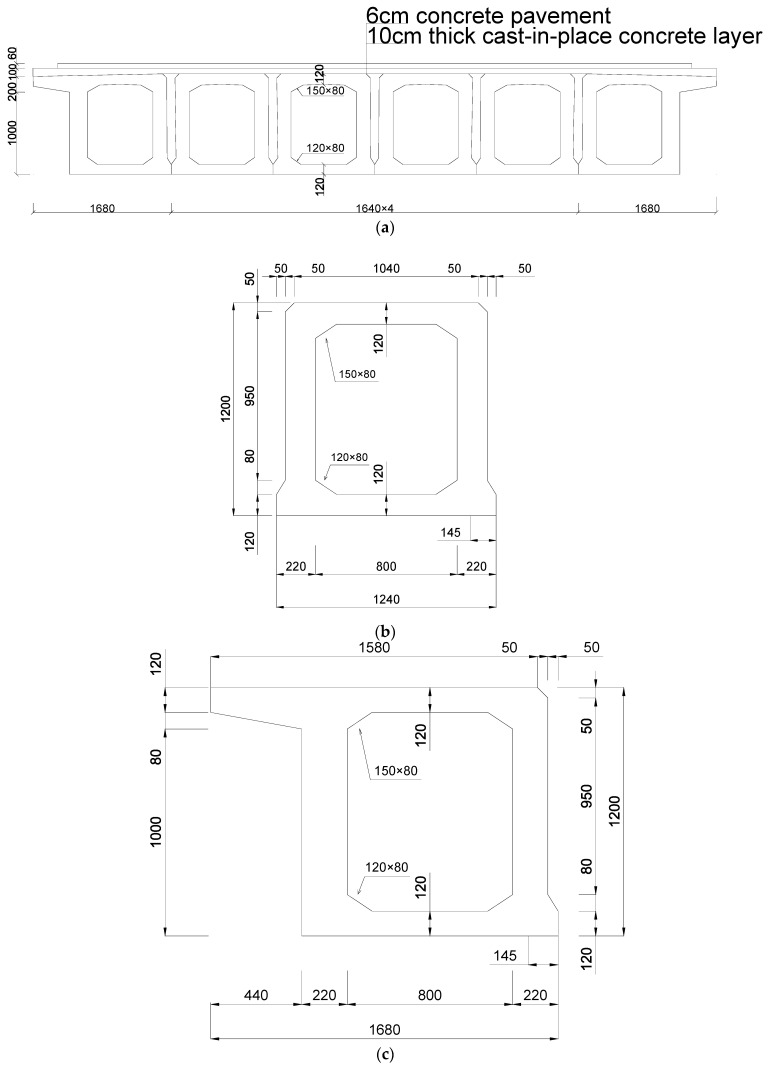
Cross section of the bridge. (**a**) Cross section of the bridge. (**b**) Cross section of the board. (**c**) Cross section ofside board.

As shown in Figure 6, the bridge structure consists of six C50 concrete box girders, a 10 cm C50 concrete levelling layer, and a 6 cm concrete pavement layer. As shown in Table A1 in the Appendix A, the bridge consists of a total of 621,502 units. The material properties of the C50 concrete are listed in Table A2 in the Appendix A.

### 3.2. Guardrail and Vehicle Model

**Figure A1:**
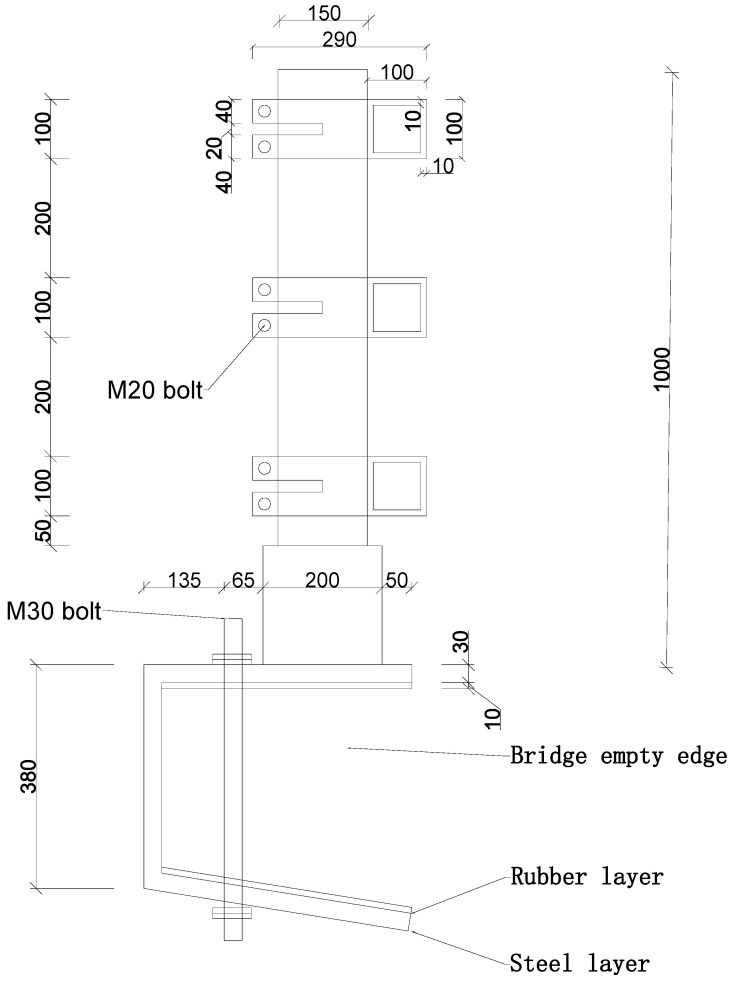
Sectional view of a guardrail.

According to the Chinese guardrail safety performance evaluation standard [26], as shown in Table A3 in the Appendix A, and in the applicable scope of a bridge, a three-level protective guardrail was designed and the specific size is shown in Figure A1 in the Appendix A [26]. The distance between the guardrail posts is 2 m, for a total of 10 m. The material properties of the steel strands and steel components of the guardrail are listed in Table A4 in the Appendix A, and the specific data of the guardrail model are listed in Table A5 in the Appendix A.

**Figure 7:**
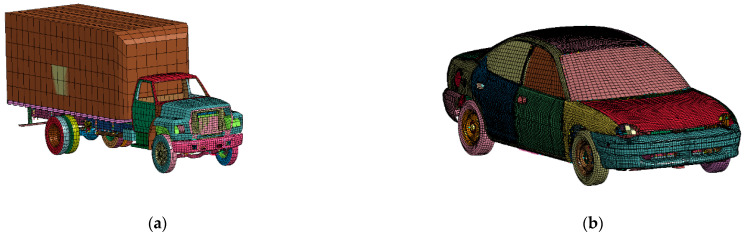
Three-dimensional view of the FE model: (**a**) a Ford single unit truck and (**b**) a Dodge Neon.

Two vehicle models were used in this study: one is Ford’s simplified model (35,353 elements) single-unit truck (SUT), and the other is a Dodge Neon (270,752 elements) (Figure 7) [27]. These models were downloaded from the NCAC website. The experimental tests include frontal collisions to verify each model [28,29]. Both models are highly correlated with the experimental results. Different vehicle speeds were investigated in this study. The vehicle’s initial speed ranged from 60 km/h to 100 km/h, and most of the vehicle’s initial speed is 100 km/h. The vehicle masses are 1.5 t for the Dodge Neon and 10 t for the Ford SUT. The quality of the Ford SUT changed when changing the quality of the goods, and the quality of the Dodge Neon changed when adjusting the quality of the engine. Table A6 in the Appendix A shows the vehicle model data and vehicle material parameters.

### 3.3. Crash Test

**Figure 8:**
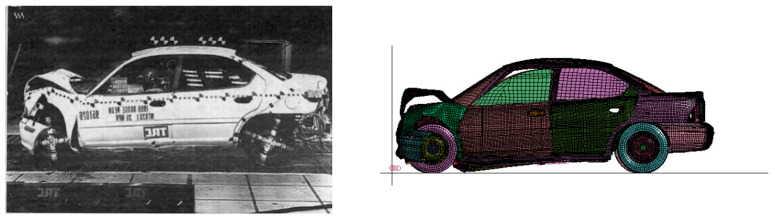
Comparison of snapshots of a frontal, rigid wall collision.

**Figure 9:**
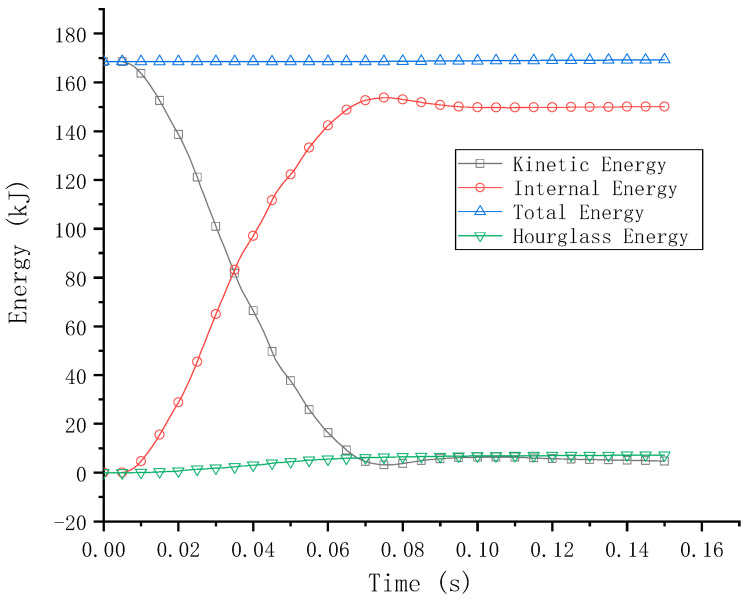
The energy–time curve of a vehicle crashing into a rigid wall.

As shown in Figure 8, in order to verify the accuracy of the vehicle–guardrail collision model, the numerical simulation between the vehicle and the rigid wall was repeated using LS-DYNA to compare the experimental collision results [28]. Figure 9 shows the energy–time curve. As shown in Figure 9, the deformed shape of the vehicle in the simulation model (on the right) is very consistent with the results of the crash test (on the left) [27]. In Figure 9, the moment at 0 s represents the start of the collision. For the vehicle model, the entire collision process lasts for 0.15 s. The hourglass energy is caused by the use of reduced integration in the display analysis. The so-called reduced integration means that the number of integration points in the calculation of the unit is less than the actual number. This operation can speed up the calculation speed, but it causes a zero energy mode of the unit, which is the hourglass energy. It can be seen from Figure 9 that the hourglass energy used to evaluate the crash performance of a car crash is only 2.3% of the initial kinetic energy, which is less than the maximum 5% of the total energy [30]. It is assumed that the accuracy of the finite element model can be guaranteed and that the simulation method of the collision process can be used in the subsequent simulations of this study.

### 3.4. Calculation Condition Setting

Different car models, different collision points, different collision angles, and different vehicle speeds were studied and compared. Eight samples, from N1 to N8, were studied, with the sample of N1 used as the reference sample. Table A7 in the Appendix A summarizes the range of selected parameters in the sample. Although several selected parameters are not common, they are used to study the performance of the new assembled guardrail under various parameters. One parameter in every group was studied when the other parameters remained unchanged, as shown in the reference sample of N1.

## 4. Guardrail Collision Performance Evaluation

**Figure 10:**
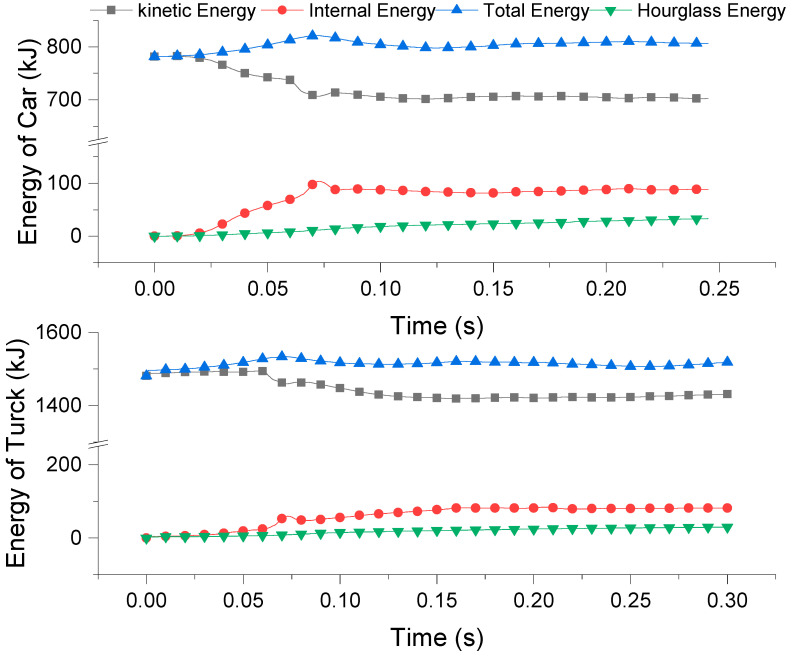
The energy–time relationship of the vehicle–ground–guardrail system.

According to Chinese regulations, the barrier collision conditions should meet the requirements in Table A3 in the Appendix A. Therefore, the results are analyzed from samples N1 and N2. When the initial kinetic energy is completely transformed into internal energy, hourglass energy, and residual kinetic energy, the finite element results are reliable [31]. The hourglass energy is calculated for every model. As shown in Figure 10, the N1 and N2 hourglass energy accounts for 4% and 2% of the total energy, respectively, which are both less than 5% of the total energy. Therefore, the hourglass control does not affect the accuracy of the results [26].

### 4.1. Energy

The energy–time curve of trucks and cars crashing into the guardrail is shown in Figure 10. As time passes, the kinetic energy of trucks and cars gradually decreases and the internal energy of the guardrail system gradually increases. The final energy conversion rates of the internal energy of the car and truck (the entire guardrail system) and the initial kinetic energy are 11.2% and 5.5%, respectively. The conversion rate of a car is twice that of a truck. This is because the initial speed of a car is larger but its mass is smaller than that of the truck. Finally, the guardrail deformation caused by a truck and a car is almost same. Therefore, the internal energy of a car and truck guardrail system is basically the same, but because the mass of the car is much smaller than that of the truck and the initial kinetic energy of the car is much smaller than that of the truck, the energy conversion rate of the car collision system is much bigger than that of the truck collision system.

From Figure 10, due to the deformability and friction of the assembled guardrail, the internal energy of the car and truck system increases steadily after collision and reaches maximum values of 103 kJ and 59 kJ at 0.075 s. Then, the kinetic energy of the car and truck system increases slightly and the internal energy decreases slightly. This is because the deformation of the steel strand is gradually restored, the internal energy of the guardrail system decreases, and the kinetic energy of the vehicle increases. At the end of the simulation, the internal energy gradually stabilizes at 88 kJ and 82 kJ.

### 4.2. Vehicle Trajectory

**Figure 11:**
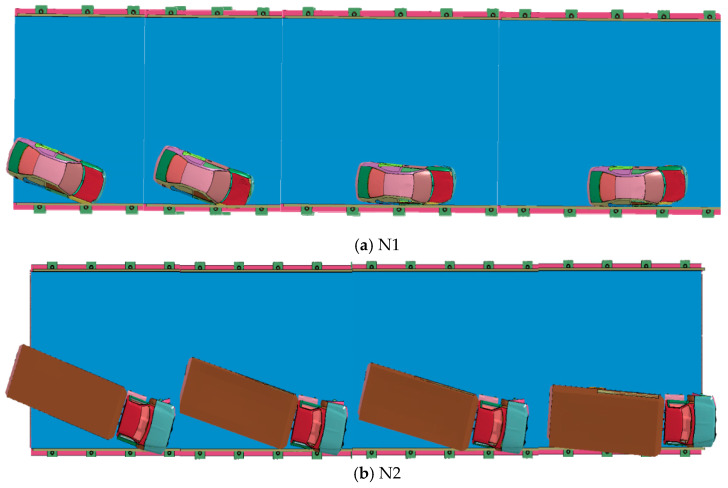
Snapshot of sample (**a**) N1, (**b**) N2 during collision.

Figure 10 shows a schematic diagram of the movement of the car guardrail after a collision. It can be seen from Figure 11 that this steel bridge guardrail has a good guiding effect for an out-of-control vehicle. After 0.14 s after the collision, the car wheels return to the correct driving direction. When a car crashes into the guardrail at a speed of 100 km/h, the car keeps moving forward and makes contact with the guardrail under the inertial force. The car starts to change its direction because of the guardrail blocks movement, causing the angle between the car and the guardrail to decrease continuously. When t is 0.15 s, the car is basically parallel to the guardrail and returns to the normal driving direction. When t is 0.175 s, the car changed its direction completely and drives away from the guardrail. The collision process of a truck hitting a movable assembled bridge guardrail is shown in Figure 11**.** The truck collision on the guardrail starts at 0.05 s, while the front of the truck starts to change its direction at 0.09 s, and then completely changes its direction at 0.17 s. The truck starts to collide with the guardrail at 0.2 s, and then, the whole truck changes its direction completely at 0.3 s.

**Figure 12:**
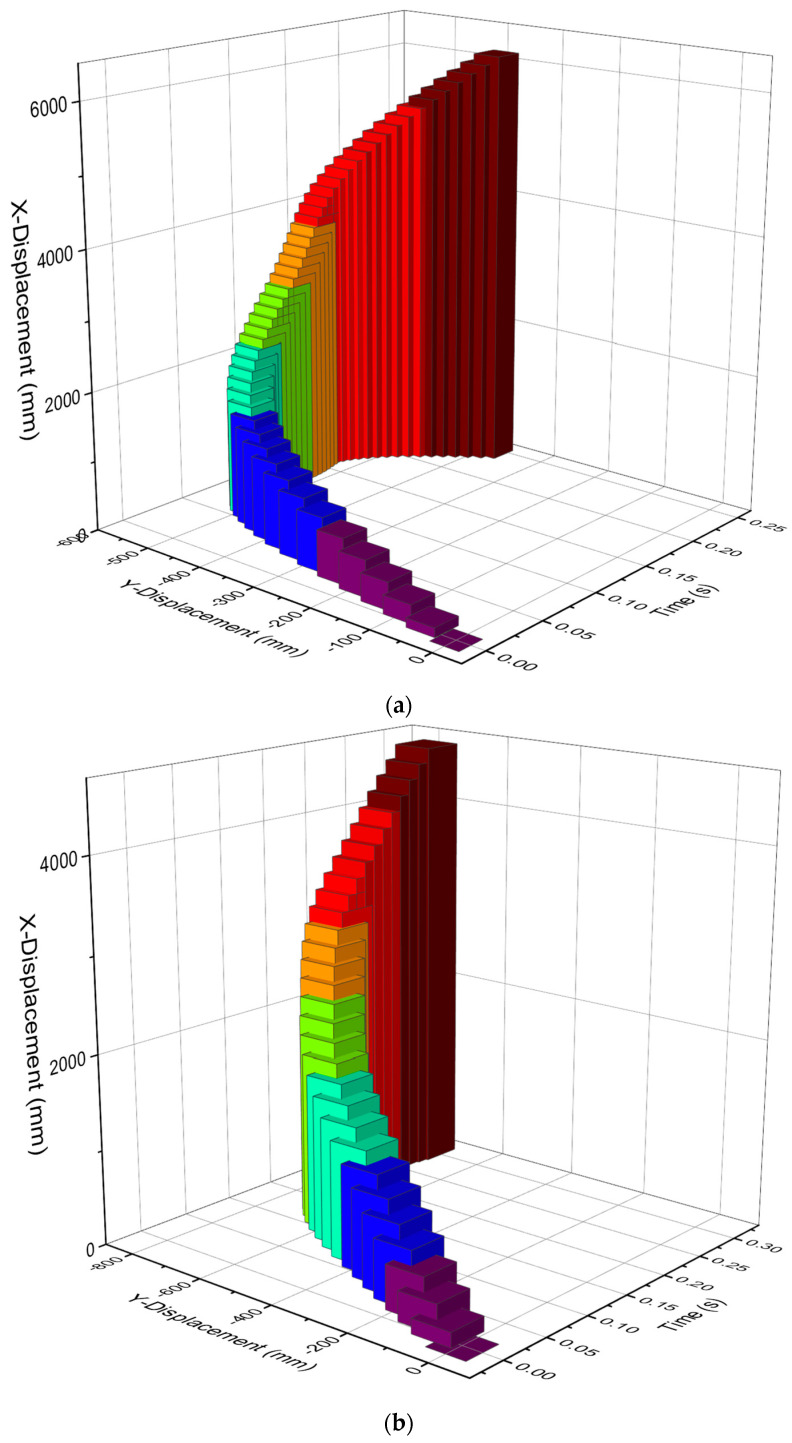
Vehicle trajectory diagram. (**a**) Car trajectory diagram. (**b**) Truck trajectory diagram.

The X axis is the longitudinal direction of the guardrail while the Y axis is the lateral direction of the guardrail in Figure 12. Figure 12 shows the displacement–time curve of a point on the left seat of the car and a point on the truck seat in the x and y directions. Figure 12 shows the trajectory of the car and the truck. It can be seen from Figure 12 that the displacement of the car in the cross-bridge direction reaches a maximum value of 554.4 mm at about 0.165 s. After the car turns, the displacement gradually decreases in the cross-bridge direction. The cross-bridge displacement of the truck reaches a maximum of 780.15 mm at about 0.23 s, and then, the truck changes its direction.

### 4.3. Stress

**Figure 13:**
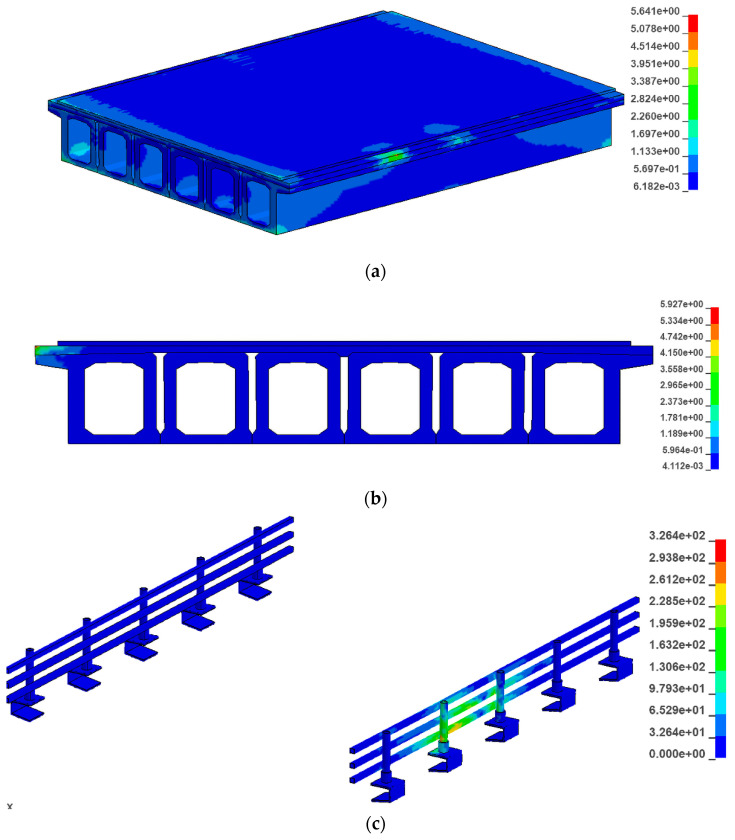

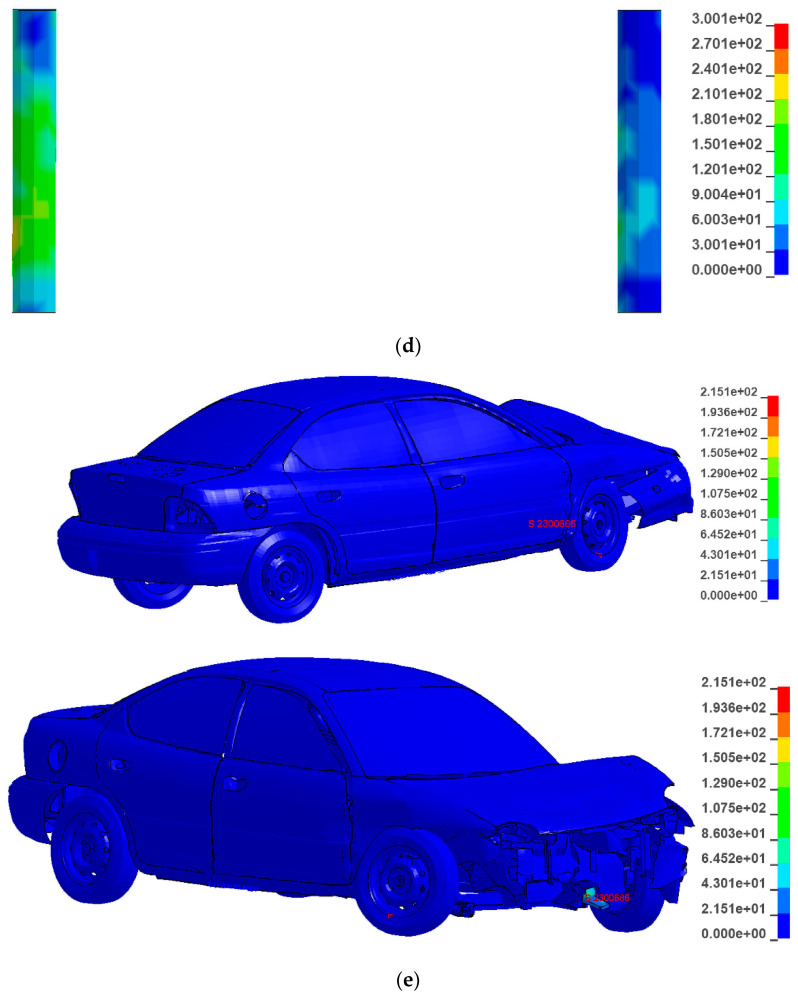
Stress cloud diagram of each main structure of sample N1. (**a**) Stress cloud diagram of the main bridge. (**b**) Stress cloud diagram of the cross section of the guardrail support at the collision point of the main bridge. (**c**) Guardrail stress cloud diagram. (**d**) Stress diagram of guardrail column in the collision area. (**e**) Car stress cloud chart.

The von Mises stress cloud diagrams of a 0.4 s collision between the main bridge, the guardrail, and the car at a speed of 100 km/h and a collision angle of 20° is shown in Figure 13. The maximum value of the main girder stress is 5.9 MPa, below the guardrail base of the collision column. The maximum value is less than the failure stress of C50 concrete, so the bridge structure is safe. As shown in Figure 13c, the maximum value of the guardrail structure stress is 326.4 MPa, which is located at the lower beam. The maximum value is less than the yield stress of 345 MPa and the ultimate strength of 470 MPa. The maximum stress of the guardrail beam at this moment is close to the yield strength, but it is less than the yield strength. Therefore, the guardrail does not reach a damaged state, and the guardrail structure is safe. Figure 13d shows the stress in the collision area of the guardrail column. The maximum stress of the column is 300 MPa, less than the steel yield stress of 345 MPa. Therefore, the guardrail column is safe.

The car stress cloud diagram is shown in Figure 13e. The maximum stress is 215.1 MPa, and the maximum stress unit is 2,303,801 units. It is located at the junction of the bumper and the car body. It does not reach the yield stress of the vehicle material, so the car deforms greatly without damage and in a safe state.

### 4.4. Guardrail Displacement

**Figure 14:**
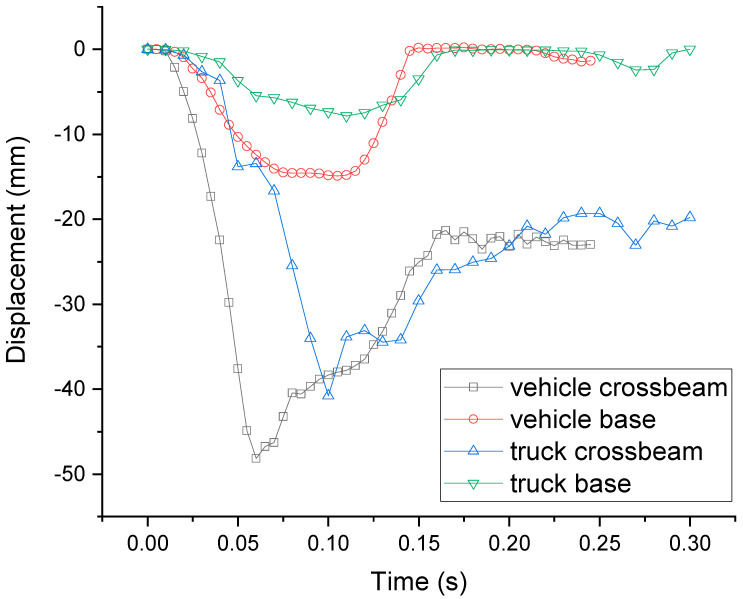
Displacement–time curve of the guardrail base and beam in the y direction (transverse bridge direction).

The lateral displacement of the guardrail is an important indicator of guardrail and vehicle safety. Taking the maximum displacement node in the guardrail beam displacement cloud map and a node of the maximum displacement base of the guardrail, the curve of the lateral displacement of the guardrail beam over time is drawn as shown in Figure 14. The maximum displacement of the saddle-shaped base of the vehicle collision guardrail is 15 mm while the maximum displacement of the guardrail beam is 49 mm and the maximum deformation of the guardrail beam is 34 mm. The maximum displacement of the saddle-shaped base of a truck is 8 mm, while the maximum displacement of the guardrail beam is 41 mm and the maximum deformation of the guardrail beam is 33 mm. They all meet the guardrail of the bridge’s maximum lateral dynamic deformation value of 0.3–0.6 m in the design code of highway traffic safety facility requirements.

### 4.5. The Survivability of the Passengers

#### 4.5.1. Vehicle Acceleration

The survival of passengers is mainly based on acceleration and the possibility of landing on the bridge.

**Figure 15:**
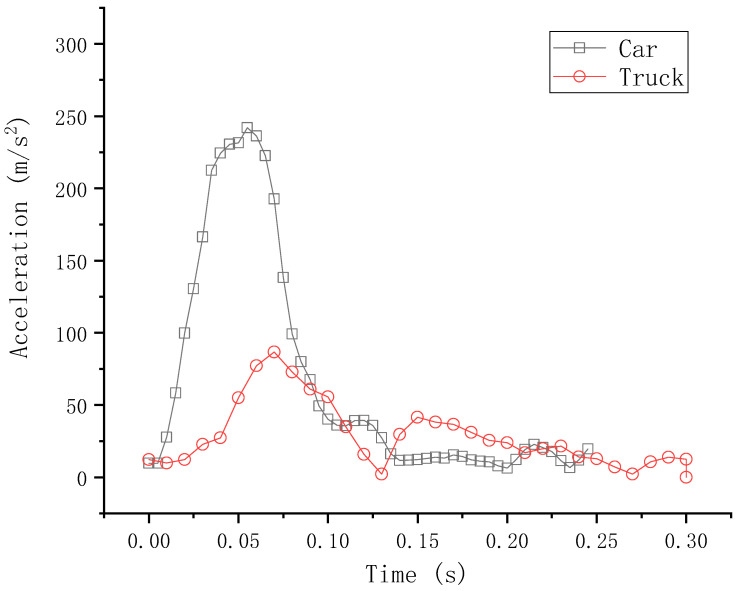
Acceleration–time curve of cars and trucks.

Vehicle acceleration is an important parameter of evaluating the survivability of passengers. Since the acceleration of each point on the car body is not same, we chose the engine of cars and trucks to draw the acceleration–time curve. Figure 15 shows the acceleration–time curve of cars and trucks. The acceleration of a car is generally less than 200 m/s^2^, but some exceed 200 m/s^2^. The peak acceleration of the truck is 87 m/s^2^, much smaller than the peak acceleration of a car as the initial speed of the truck is 60 km/h, much smaller than that of the car (100 km/h). This shows that the guardrail is well-oriented and that the passengers are relatively uninjured.

#### 4.5.2. Vertical Rise of the Vehicle’s Center of Gravity 

**Figure 16:**
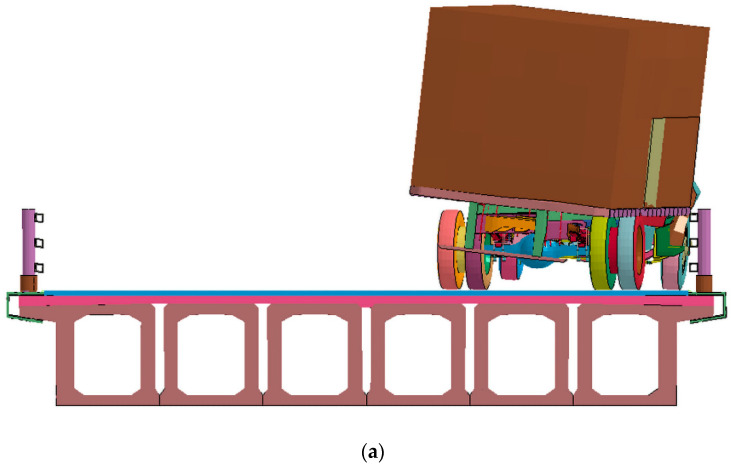

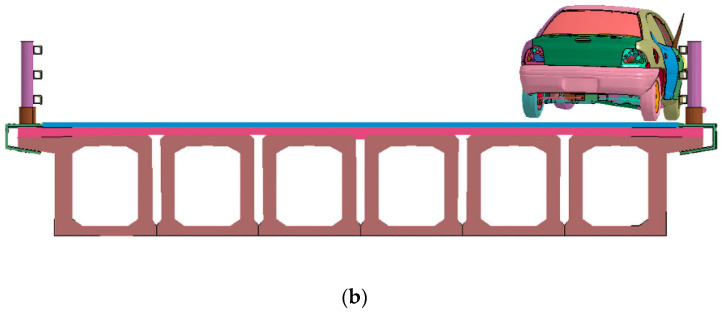
Tilting diagram of the assembled guardrail and car: (**a**) truck tilt chart and (**b**) car tilt chart.

As shown in Figure 16, during the collision process, the truck and car are slightly tilted, with no danger of overturning. Therefore, the vehicle and passengers are safe and the guardrail has good guidance performance.

#### 4.5.3. Safety of the Guardrail Structure

It can be seen from Section 4.1 and Section 4.2 that the guardrail structure is in a safe state. The guardrail structure is not damaged, and the car does not fall from the bridge.

### 4.6. Bridge Damage

**Figure 17:**
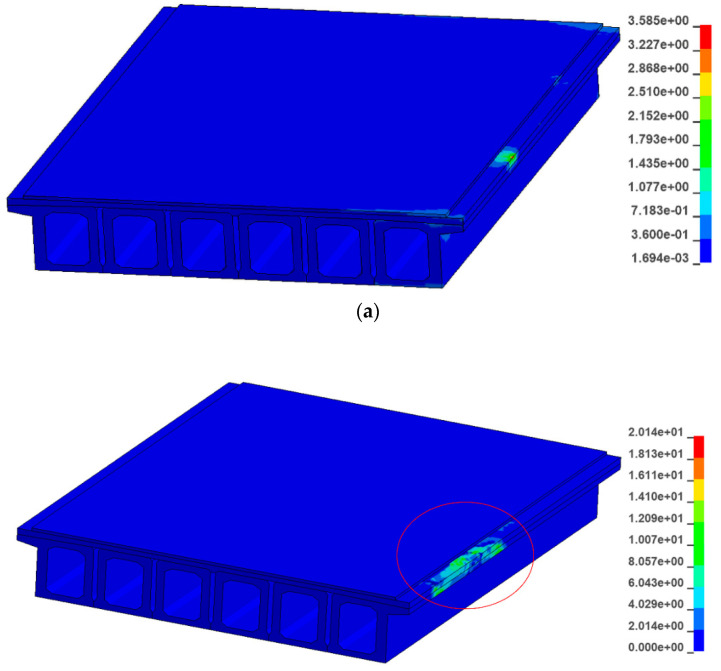

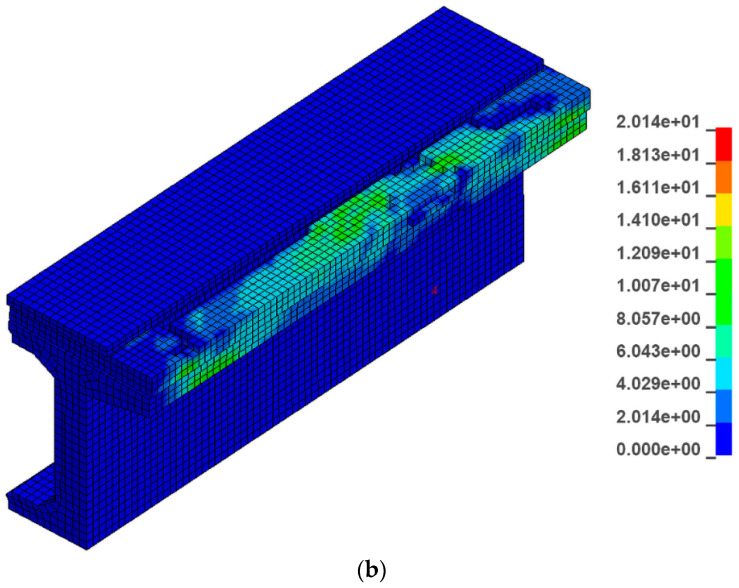
Maximum tensile stress diagram: (**a**) fabricated guardrail and (**b**) traditional guardrail.

**Figure 18:**
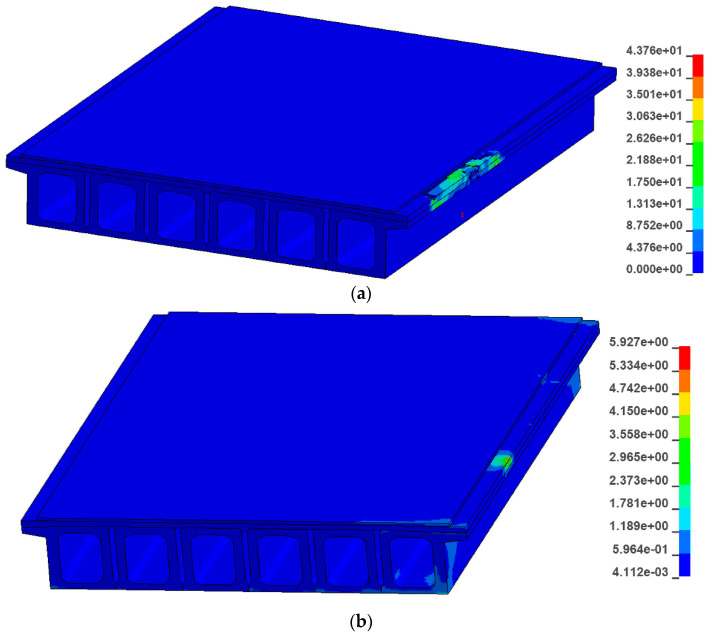
V-M stress diagram: (**a**) fabricated guardrail and (**b**) traditional guardrail.

In order to study the damage caused by the collision between the car and the guardrail to the bridge, the ultimate stress failure of concrete and steel is defined in LS-DYNA. It can be seen from the collision force–time curve that the collision force is the largest at 0.04 s. Figure 17 shows the maximum tensile stress diagram of the fabricated guardrail and the traditional guardrail at 0.04 s; Figure 18 shows the V-M stress diagram of the fabricated guardrail and the traditional guardrail at 0.04 s.

It can be observed that the main beam of the traditional guardrail is more severely damaged than the main beam of the prefabricated guardrail. This is because the traditional guardrail is embedded in the concrete of the main beam. When the car collides with the guardrail, the main beam structure is destroyed while, for the prefabricated guardrail, the structure is not embedded in the bridge structure, so the main girder is not damaged. It can be seen from the figure that the maximum tensile stress of the main beam of the traditional guardrail structure is 20.1 MPa, which is much greater than the 3.6 MPa of the fabricated guardrail. The concrete main beam of the traditional guardrail structure is damaged by tension. The maximum V-M stress of the traditional guardrail structure is 43.8 MPa, which is much larger than the 5.9 MPa of the fabricated guardrail structure.

## 5. Impact of Crash Parameters on Collision Performance

### 5.1. Result Analysis

**Figure 19:**
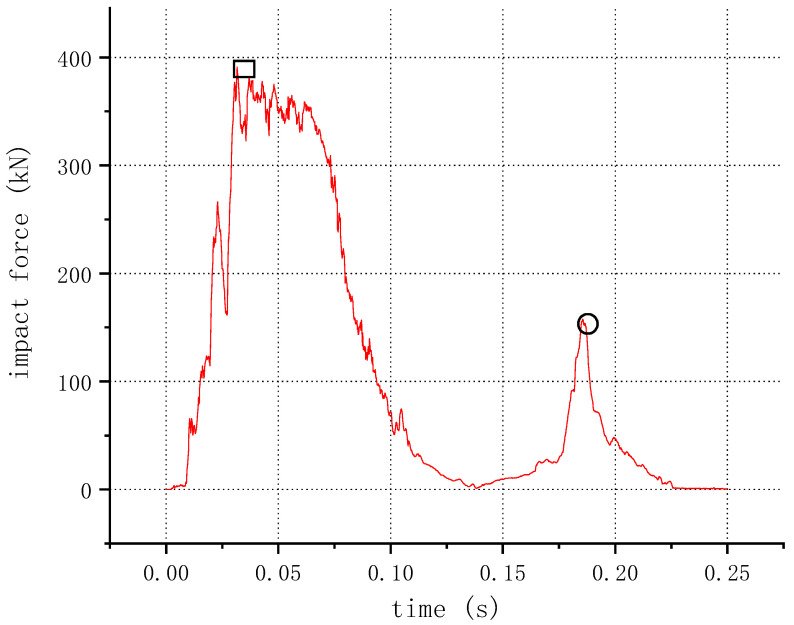
Time–impact force of sample N1. □: a front impact; ○: a tail impact.

The time–impact force relationship of the assembled bridge guardrail in a vehicle collision is shown in Figure 19. The peak dynamic force (PDF) was investigated. The first peak force appears when the front of the vehicle collides with the guardrail. The second peak force on the guardrail is caused by the collision between the car’s rear and the guardrail. Each sample reaches its PDF almost at the same time (32–50 ms), and the impact force is 0 after 230 ms. Samples N3 and N7 only have one peak force each as the initial speed of sample N3 is small and the rear of the vehicle moved away without crashing into the guardrail. Since the collision angle of sample N7 is small and the vehicle turns easily, the rear of the vehicle does not collide with the guardrail. Table A8 in the Appendix A summarizes the PDF and the maximum deformation of the guardrail beam of all samples. The maximum deformation of the guardrail beam is 73 mm. According to the literature [28], the transverse deformation Z of the bridge beam–column steel guardrail ranges from 0.3 m to 0.6 m, which meet the requirements.

**Figure 20:**
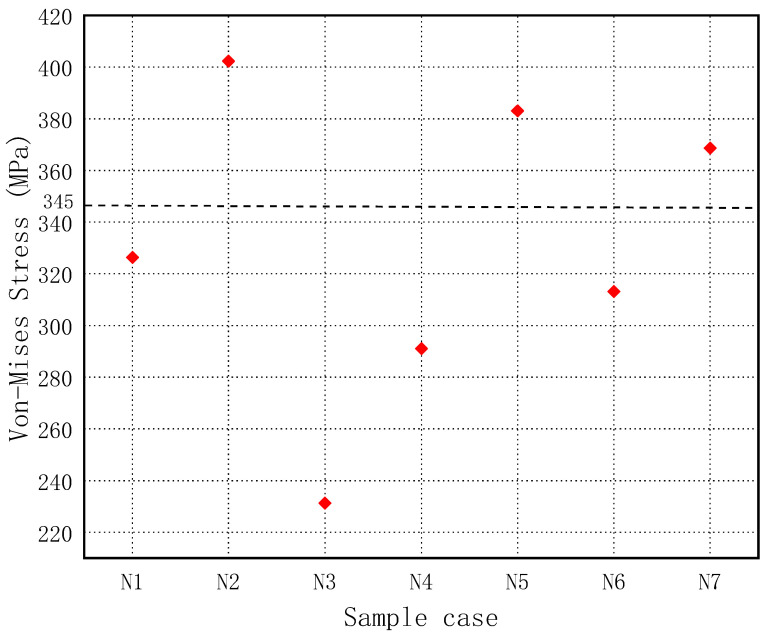
Maximum von Mises stress of the guardrail.

If the maximum von Mises stress of the guardrail structure reaches the maximum strength of steel, the guardrail structure is locally damaged. Therefore, the maximum von Mises stress of the guardrail structure is vital to the safety of vehicles and passengers. Figure 20 shows the maximum von Mises stresses of all sample guardrail structures. All sample guardrails did not reach the maximum stress of steel at 470 MPa, but samples N2, N5, and N7 reached the yield stress of the guardrail material at 325 MPa. After yielding, the guardrail structure is in a safe state. 

### 5.2. Vehicle Quality

**Figure 21:**
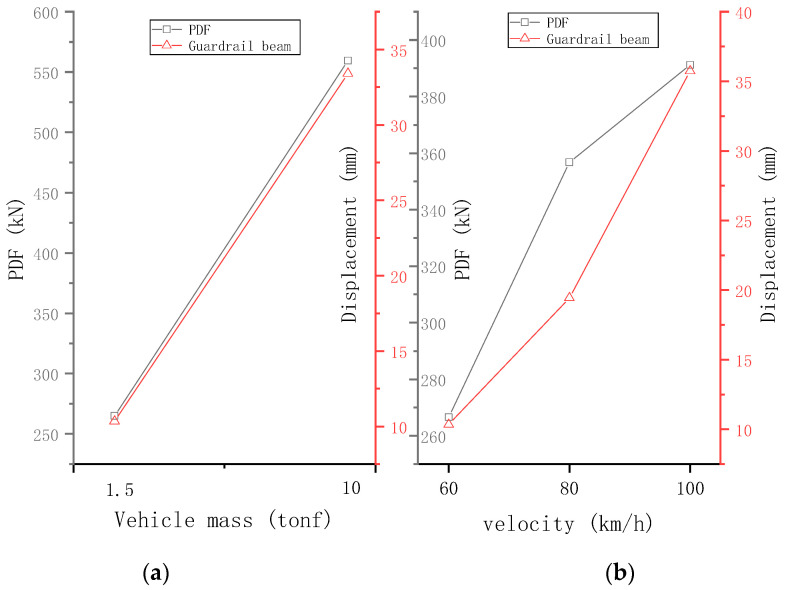
Effects of (**a**) vehicle velocity and (**b**) vehicle mass on PDF and maximum displacement of the guardrail beam.

**Figure 22:**
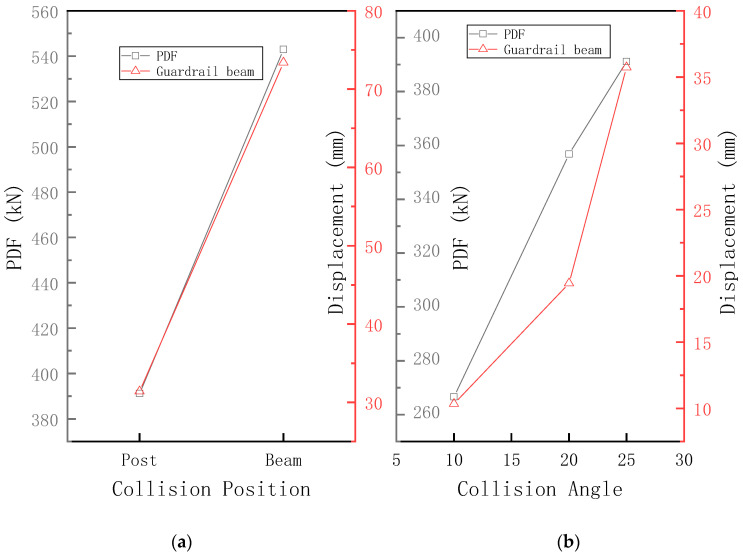
Effects of (**a**) collision point and (**b**) collision angle on PDF and maximum displacement of the guardrail beam.

Two vehicles with masses of 1.5 t and 10 t were studied. The PDF and maximum dis-placement of guardrail beam reflect the severity of the collision and the safety of the guardrail. Figure 21 shows the effects of vehicle velocity and vehicle mass on PDF and maximum displacement of the guardrail beam. Figure 22a shows the PDF of samples with different vehicle masses and the maximum lateral deformation of the guardrail beam. When a vehicle’s mass increases, the PDF and the lateral deformation of the maximum guardrail beam increase linearly. When vehicle’s mass increases by 5.7 times, the PDF increases by 111% and the maximum deformation of the guardrail beam increases by 230%.

### 5.3. Vehicls Speed

Three vehicle speeds were studied, ranging from 60 km/h to 100 km/h. Figure 21b shows the PDF and the maximum lateral deformation of the guardrail beam with different vehicle speed samples. When the vehicle speed increases, the PDF tends to increase nonlinearly. The increase in the PDF is not proportional to the square of the velocity, similar to the elastic collision problem. The damage to the guardrail reduces the growth rate of PDF. When the vehicle speed increases from 60 km/h to 100 km/h, the PDF increases by approximately 46.7%. The increase in the maximum lateral deformation of the guardrail beam is roughly proportional to the square of the speed. When the vehicle speed increases from 60 km/h to 80 km/h, the square of the speed increases by 11.1% and the maximum lateral deformation of the guardrail beam increases by 88%. When the speed increases from 80 km/h to 100 km/h, the square of the speed increases by 6.3% and the maximum lateral deformation of the guardrail beam increases by 83.8%. The rate of increase in the deformation of the guardrail beam is slightly greater than the former because the greater the speed, the more severe the damage of the guardrail and the faster the guardrail deforms.

### 5.4. Collision Location

Two collision locations were studied: the middle of the guardrail beam and the column. Figure 22b shows sample PDFs at different collision locations and the maximum lateral deformation of the guardrail beam. When the collision point changes from the guardrail column to the middle of the guardrail beam, the maximum lateral deformation of the PDF and the guardrail beam increases by 38.8% and 134%, respectively. When the collision occurs at the column, the steel strand of the column base deforms, which can reduce the PDF and the maximum deformation of the guardrail beam effectively. 

### 5.5. Collision Angle

Three collision angles of 10°, 20°, and 25° were studied. Figure 22a shows the PDF of samples with different collision angles and the maximum lateral deformation of the guardrail beam. The PDF tends to increase nonlinearly with increasing collision angle. The increase in PDF is not proportional to the angle, which is similar to an elastic collision. The damage of the guardrail reduces the growth rate of PDF. When the collision angle increases from 10° to 25°, the PDF increases by 127%. With a larger collision angle, the vehicle experiences more difficulty in turning and the collision force becomes greater. When the collision angle increases, the maximum lateral deformation of the guardrail beam increases nonlinearly: the larger the angle, the faster the growth rate increases. Hence, if the collision angle increases, the guardrail is damaged partially and the maximum lateral deformation of the guardrail beam increases rapidly.

When the collision angle is 25 degrees, the 1.5 t car collides with the guardrail at 100 km/h, the U-shaped base at the collision point is partially damaged, and the guardrail structure as a whole maintains a safe state. In order to ensure the safety of passengers, when the collision angle is 25 degrees, the speed of the 1.5 t car cannot exceed 80 km/h.

## 6. Conclusions

This paper proposes a new type of prefabricated bridge guardrail. Through comprehensive parameter research, the impact effects of vehicles and prefabricated bridge guardrails are evaluated. The two main terms used in this study of vehicle collisions with prefabricated bridge guardrails are peak dynamic force (PDF) and maximum lateral deformation of the guardrail beam. This comprehensive parameter research examines the influence of car mass, collision angle, initial vehicle speed, and collision point on the collision effect. Here are the conclusions:

(1) When the mass of the car increases, PDF and the lateral displacement of the guardrail beam increase significantly. When vehicle’s mass increases by 5.7 times, the PDF increases by 111% and the maximum deformation of the guardrail beam increases by 230%.
(2) When the vehicle speed increases, the PDF tends to increase nonlinearly. The increase in PDF is not proportional to the square of the velocity, and the increase in the maximum lateral deformation of the guardrail beam is roughly proportional to the square of the speed.
(3) When the collision point changes from the guardrail column to the middle of the guardrail beam, the maximum lateral deformation of the PDF and the guardrail beam increase by 38.8% and 134%, respectively.
(4) The PDF and the maximum lateral deformation of the guardrail beam tend to increase nonlinearly with increasing collision angle. When the collision angle becomes larger, the speed of PDF becomes slower. When the collision angle increases, the guardrail is damaged partially and the maximum lateral deformation of the guardrail beam increases rapidly.

In summary, the new type of prefabricated bridge guardrail can meet the recommended safety performance evaluation criteria and has good crash performance. The limitations of the current study and future research directions are given as follows:

(1) The appropriate range for guardrail height should be determined according to the road grade.
(2) The diameter or distance between steel strands should be adjusted to increase the displacement of the U-shaped base and to further reduce vehicle acceleration.

## Data Availability

Not applicable.

## Appendix A

**Table A1:** Data of the bridge mode.

Model		Bridge
Number of elements	Solid	621502
	Total	621502
Weigh (t)		126.16

**Table A2:** Bridge material (C50 concrete).

Type	Value
Density (Kg/m^3^)	2420
Poisson ratio	0.2
Compressive strength (MPa)	50
Tensile strength (MPa)	5

**Table A3:** Test collision conditions of a standard guardrail section, a transitional guardrail section, and central partition with an opening guardrail.

Protection Level	Collision Model	Vehicle Quality	Collision Velocity (km/h)	Collision Angle
One	Minibus	1.5	50	20
	Medium Bus	6	40	20
	Medium truck	6	40	20
Two	Minibus	1.5	60	20
	Medium Bus	10	40	20
	Medium truck	10	40	20
Three	Minibus	1.5	100	20
	Medium Bus	10	60	20
	Medium truck	10	60	20
Four	Minibus	1.5	100	20
	Medium Bus	10	80	20
	Medium truck	18	60	20

**Table A4:** Material properties used in the finite element guardrail model.

Component	Type	Unit	Default Value
Steel	Density	Kg/m^3^	7.85 × 10^3^
	Elastic Modulus	MPa	2.06 × 10^5^
	Poisson ratio	/	0.29
	Yield strength	MPa	345
	Ultimate strength	MPa	470
Stranded wire	Density	Kg/m^3^	7.85 × 10^3^
	Elastic Modulus	MPa	1.95 × 10^5^
	Nominal tensile strength	MPa	1860
	0.2%Yield force	kN	246

**Table A5:** Data of the guardrail model.

Model		Guardrail
Number of elements	Solid	108,374
	Beam	465
	Total	108,839

**Table A6:** Vehicle model data.

**Model**		**Car**
Number of elements	Shell	267,786
	Solid	2836
	Beam	122
	Discrete	8
	Total	270,752
Weigh (t)		1.5
Yield stress (MPa)		400
Young’s modulus (MPa)		210,000
**Model**		**Truck**
Number of elements	Beam	548
	Discrete	58
	Shell	31,557
	Solid	886
	Total	33,049
Weigh (t)		10
Yield stress (MPa)		270
Young’s modulus (MPa)		205,000

**Table A7:** Working conditions table.

Parameter		Sample Case	Collision Conditions			
			Quality (t)	Position	Velocity (km/h)	Angle
Vehicle mass	1.5	N3	1.5	post	60	20
	10	N2	10	post	60	20
Velocity	60	N3	1.5	post	60	20
	80	N4	1.5	post	80	20
	100	N1	1.5	post	100	20
Position	post	N1	1.5	post	100	20
	beam	N5	1.5	beam	100	20
Collision Angle	10	N6	1.5	post	100	10
	20	N1	1.5	post	100	20
	25	N7	1.5	post	100	30

**Table A8:** Summary of the FE results.

Variables		Sample Case	PDF (kN)	Maximum Displacement of Guardrail Beam
Parameter	Value			
Vehicle mass (t)	1.5	N1	391.17	10.34
	10	N2	559.44	33.4
Velocity	60	N3	266.63	10.34
	80	N4	356.78	19.44
	100	N1	391.17	35.74
Position	Beam	N5	542.95	73.41
	Post	N1	391.17	31.41
Collision Angle	10	N6	241.71	31
	20	N1	391.17	36
	25	N7	548.54	72

## References

[1] Qiao X., Zhang G., Wang C., Ren C. (2013). Occupant risk assessment method based on vehicle-guardrail collision. Highw. Traffic Technol..

[2] Atahan A.O., Erdem M.M. (2016). Evaluation of 12 m Long Turned Down Guardrail End Terminal Using Full-Scale Crash Testing and Simulation. Lat. Am. J. Solids Struct..

[3] Bank L.C., Yin J., Gentry T.R. (1998). Pendulum Impact Tests on Steel W-Beam Guardrails. J. Transp. Eng..

[4] Driemeier L., Yoneda A., Moura R.T., Alves M. (2016). Performance of metallic defences submitted to vehicle impact. Int. J. Crashworthiness.

[5] Gabauer D.J., Kusano K.D., Marzougui D., Opiela K., Gabler H.C. (2010). Pendulum testing as a means of assessing the crash performance of longitudinal barrier with minor damage. Int. J. Impact Eng..

[6] Neves R.R., Fransplass H., Langseth M., Driemeier L., Alves M. (2018). Performance of some basic types of road barriers subjected to the collision of a light vehicle. J. Braz. Soc. Mech. Sci. Eng..

[7] Reid J.D., Kuipers B.D., Sicking D.L., Faller R.K. (2009). Impact performance of W-beam guardrail installed at various flare rates. Int. J. Impact Eng..

[8] Sheikh N.M., Bligh R.P., Bullard D.L., Buth C.E., Alberson D.C., Abu-Odeh A.Y., Ross H.E. Development of an Energy Absorbing End Terminal for Open Box Beam Guardrail. Proceedings of the 9th International LSDYNA Users Conference.

[9] Whitworth H.A., Bendidi R., Marzougui D., Reiss R. (2004). Finite element modeling of the crash performance of roadside barriers. Int. J. Crashworthiness.

[10] Noh M.H., Lee S.Y. (2017). Construction tolerance effects of reinforced posts on crash performances of an open-type guardrail system. Thin Walled Struct..

[11] Park H., Ahn K. (2014). Behavior Analysis of Fill Slope by Vehicle Collision on Guardrail. J. Korean Geoenvironmental Soc..

[12] Wu W., Thomson R. (2007). A study of the interaction between a guardrail post and soil during quasi-static and dynamic loading. Int. J. Impact Eng..

[13] Roadside Design Guide (4th Edition; Incorporating Errata September 2011; Errata January 2012; Errata February 2012 and Errata July 2015). In US-AASHTO: 2011; Vol. AASHTO RSDG-4-2011. https://downloads.transportation.org/RSDG-4-Errata.pdf.

[14] Sicking D. (2009). Manual for Assessing Safety Hardware.

[15] Atahan A.O., Bonin G., Cicinnati L., Yasarer I.H. (2008). Development of European End-Treatment TWINY Using Simulation and Crash Testing. J. Transp. Eng..

[16] Atahan A.O., Hiekmann J.M., Himpe J., Marra J. (2017). Development of a continuous motorcycle protection barrier system using computer simulation and full-scale crash testing. Accid. Anal. Prev..

[17] Atahan A.O. (2018). Development of a Heavy Containment Level Bridge Rail for Istanbul. Lat. Am. J. Solids Struct..

[18] Yin H., Fang H., Wang Q., Wen G. (2016). Design optimization of a MASH TL-3 concrete barrier using RBF-based metamodels and nonlinear finite element simulations. Eng. Struct..

[19] Yin H., Xiao Y., Wen G., Fang H. (2017). Design optimization of a new W-beam guardrail for enhanced highway safety performance. Adv. Eng. Softw..

[20] Atahan A.O. (2006). Finite-Element Crash Test Simulation of New York Portable Concrete Barrier with I-Shaped Connector. J. Struct. Eng..

[21] Gover R.B., Oloyede A., Thambiratnam D.P., Thiyahuddin M.I., Morris A. (2015). Experimental and numerical study of polymeric foam efficacy in portable water filled barriers. Int. J. Impact Eng..

[22] Thiyahuddin M.I., Gu Y.T., Thambiratnam D.P., Thilakarathna H.M. (2014). Impact and energy absorption of portable water-filled road safety barrier system fitted with foam. Int. J. Impact Eng..

[23] Thiyahuddin M.I., Thambiratnam D.P., Gu Y.T. (2014). Effect of joint mechanism on vehicle redirectional capability of water-filled road safety barrier systems. Accid. Anal. Prev..

[24] JTG/T D81 (2017). Design Guidelines for Highway Safety Facilities.

[25] GB50010 (2010). Code for Design of Concrete Structures.

[26] (2013). JTG B05-01-2013. Standard for Safety Performance Evaluation of Highway Barriers.

[27] National Crash Analysis Center (NCAC) (1994). Public Finite element Model Archive. www.ncac.gwu.edu/archives/model/index.html.

[28] Eskandarian A., Bedewi N.E. (1994). National Crash Analysis Center. Public Roads.

[29] Chawla A., Mukherjee S., Mohan D., Bose D., Rawat P., Sakurai M., Nakatani T. (2005). FE Simulations of Motorcycle-Car Frontal Crashes, Validation and Observations. Int. J. Crashworthiness.

[30] Riazi S., Feizi M.M., Hosseini-Tehrani P. (2012). Improving Crashworthiness in Railcar Against Rollover. Trans. Can. Soc. Mech. Eng..

[31] El-Tawil S., Severino E., Fonseca P. (2005). Vehicle Collision with Bridge Piers. J. Bridge Eng..

